# Patient and Clinician Decision Support to Increase Genetic Counseling for Hereditary Breast and Ovarian Cancer Syndrome in Primary Care: A Cluster Randomized Clinical Trial

**DOI:** 10.1001/jamanetworkopen.2022.22092

**Published:** 2022-07-18

**Authors:** Rita Kukafka, Samuel Pan, Thomas Silverman, Tianmai Zhang, Wendy K. Chung, Mary Beth Terry, Elaine Fleck, Richard G. Younge, Meghna S. Trivedi, Julia E. McGuinness, Ting He, Jill Dimond, Katherine D. Crew

**Affiliations:** 1 Department of Biomedical Informatics, Vagelos College of Physicians and Surgeons, Columbia University Irving Medical Center, New York, New York; 2 Herbert Irving Comprehensive Cancer, Columbia University Irving Medical Center, New York, New York; 3 Department of Sociomedical Sciences, Mailman School of Public Health, Columbia University Irving Medical Center, New York, New York; 4 Department of Pediatrics and Medicine, Columbia University Irving Medical Center, New York, New York; 5 Department of Epidemiology, Columbia University Irving Medical Center, New York, New York; 6 Division of Community and Population Health, New York Presbyterian Hospital, New York; 7 Department of Medicine, Vagelos College of Physicians and Surgeons, Columbia University Irving Medical Center, New York, New York; 8 Department of Biomedical Informatics, Johns Hopkins University, Baltimore, Maryland; 9 Sassafras Collective, Ann Arbor, Michigan

## Abstract

**Importance:**

To promote the identification of women carrying *BRCA1/2* variants, the US Preventive Services Task Force recommends that primary care clinicians screen asymptomatic women for an increased risk of carrying a *BRCA1/2* variant risk.

**Objective:**

To examine the effects of patient and clinician decision support about *BRCA1/2* genetic testing compared with standard education alone.

**Design, Setting, and Participants:**

This clustered randomized clinical trial was conducted at an academic medical center including 67 clinicians (unit of randomization) and 187 patients. Patient eligibility criteria included women aged 21 to 75 years with no history of breast or ovarian cancer, no prior genetic counseling or testing for hereditary breast and ovarian cancer syndrome (HBOC), and meeting family history criteria for *BRCA1/2* genetic testing.

**Interventions:**

RealRisks decision aid for patients and the Breast Cancer Risk Navigation Tool decision support for clinicians. Patients scheduled a visit with their clinician within 6 months of enrollment.

**Main Outcomes and Measures:**

The primary end point was genetic counseling uptake at 6 months. Secondary outcomes were genetic testing uptake at 6 and 24 months, decision-making measures (perceived breast cancer risk, breast cancer worry, genetic testing knowledge, decision conflict) based upon patient surveys administered at baseline, 1 month, postclinic visit, and 6 months.

**Results:**

From December 2018 to February 2020, 187 evaluable patients (101 in the intervention group, 86 in the control group) were enrolled (mean [SD] age: 40.7 [13.2] years; 88 Hispanic patients [46.6%]; 15 non-Hispanic Black patients [8.1%]; 72 non-Hispanic White patients [38.9%]; 35 patients [18.9%] with high school education or less) and 164 (87.8%) completed the trial. There was no significant difference in genetic counseling uptake at 6 months between the intervention group (20 patients [19.8%]) and control group (10 patients [11.6%]; difference, 8.2 percentage points; OR, 1.88 [95% CI, 0.82-4.30]; *P* = .14). Genetic testing uptake within 6 months was also statistically nonsignificant (13 patients [12.9%] in the intervention group vs 7 patients [8.1%] in the control group; *P* = .31). At 24 months, genetic testing uptake was 31 patients (30.7%) in intervention vs 18 patients (20.9%) in control (*P* = .14). Comparing decision-making measures between groups at baseline to 6 months, there were significant decreases in perceived breast cancer risk and in breast cancer worry (standard mean differences = −0.48 and −0.40, respectively).

**Conclusions and Relevance:**

This randomized clinical trial did not find a significant increase in genetic counseling uptake among patients who received patient and clinician decision support vs those who received standard education, although more than one-third of the ethnically diverse women enrolled in the intervention underwent genetic counseling. These findings suggest that the main advantage for these high-risk women is the ability to opt for screening and preventive services to decrease their cancer risk.

**Trial Registration:**

ClinicalTrials.gov Identifier: NCT03470402

## Key Points

**Question:**

What is the effect of patient and clinician decision support on genetic counseling uptake among women screened in the primary care setting for hereditary breast and ovarian cancer syndrome genetic testing eligibility?

**Findings:**

In this randomized clinical trial of 187 women and 67 clinicians, genetic counseling uptake was 19.8% for patients who received the decision aid vs 11.6% patients who did not, although this was not a statistically significant difference.

**Meaning:**

In this randomized clinical trial, there was not a significant increase in genetic counseling uptake, although more than one-third of the ethnically diverse women enrolled to the intervention underwent genetic counseling.

## Teaser

This cluster randomized clinical trial investigates the effects of patient and clinician decision support for *BRCA1/2* genetic testing compared with standard education alone.

## Introduction

Up to 10% of breast cancers^1,2,3,4,5,6^ and 15% to 20% of ovarian cancers^5,6^ are attributed to high-risk pathogenic variants in cancer susceptibility genes and are therefore potentially preventable.^7,8,9^ Women with hereditary breast and ovarian cancer syndrome (HBOC) attributable to *BRCA1* and *BRCA2* (*BRCA1/*2) variants have a lifetime breast cancer risk of 60% to 80% and a lifetime ovarian cancer risk of 20% to 40%.^10,11,12^ Identifying women with *BRCA1/2* variations can inform risk management and prevention strategies, including intensive breast cancer screening with mammography and breast MRI,^13,14,15^ risk-reducing surgeries (prophylactic mastectomy, bilateral salpingo-oophorectomy [BSO]),^16,17,18,19,20,21,22^ and chemoprevention.^23,24^ Such preventive strategies can reduce a *BRCA1/*2 carrier’s cancer risk by up to 90% once she is identified.^25^

To promote the identification of women carrying *BRCA1/2* variants, the United States Preventive Services Task Force (USPSTF) recommends that primary care clinicians screen asymptomatic women for an increased risk of *BRCA1/2* variants.^9,26^ Women who screen positive should receive genetic counseling by a trained clinician and be offered *BRCA1/2* testing if further indicated and desired after counseling.^26^ The identification of women whose family history indicates an increased risk for carrying a variant is based upon a set of risk factors including early onset of breast or ovarian cancer, multiple cases of breast or ovarian cancer in the family, bilateral breast cancer, male breast cancer, Ashkenazi Jewish descent, or a previously identified *BRCA1/2* variant in the family.^27^

Despite the USPSTF recommendation and the increasing availability of genetic testing, many women at an increased risk of carrying *BRCA1/2* variants are never identified and are thus unable to receive downstream preventive services.^28,29,30,31,32^ Although the prevalence of *BRCA1/2* variants is similar across most racial and ethnic groups (except Ashkenazi Jewish individuals), women in minoritized ethnic and racial groups and lower education and income levels are less likely to be referred for genetic testing.^28,33,34^ This lack of risk assessment in minoritized racial and ethnic populations can widen health disparities and poorer clinical outcomes.^35^

Clinicians find it difficult to assess breast cancer risk and communicate probabilistic risk information to their patients during the primary care encounter.^36^ Barriers to family history screening and genetic counseling referral include insufficient knowledge of HBOC and inability to estimate risk,^37,38,39,40,41^ lack of time and competing priorities in the primary care encounter,^36,42^ and inadequate reporting of family history in medical records.^32,43^ Patient barriers to discussing and understanding risk include lack of knowledge, low health literacy or numeracy, language barriers, and time constraints.^36,44,45^

To address these issues, we developed the RealRisks decision aid (DA) for women to screen for HBOC eligibility and the Breast Cancer Risk Navigation Tool (BNAV) for their clinicians.^46,47^ Both tools were designed by our study team using participatory design methods and pilot tested among ethnically diverse women at high risk for breast cancer.^36,48,49,50^ This cluster randomized clinical trial compared the effectiveness of RealRisks and BNAV to patient education alone in promoting appropriate uptake of *BRCA1/2* genetic counseling. We hypothesized that exposure to RealRisks and BNAV would lead to increased uptake of genetic counseling compared with patient education alone.

## Methods

### Study Design

The BEATRICE Study was an unblinded cluster randomized clinical trial that compared the effectiveness of RealRisks and BNAV, combined with standard educational materials with a control group of standard educational materials alone with clustering at the clinician level. For more details, the trial protocol is previously described.^51^ The study followed the Consolidated Standards of Reporting Trials (CONSORT) reporting guideline. The study protocol can be found in Supplement 1. Study procedures (eFigure in Supplement 2) were approved by the Columbia University Irving Medical Center (CUIMC) institutional review board.

### Setting and Participants

Participants were recruited from the outpatient clinics of CUIMC in New York City, providing a range of services, including internal medicine, family medicine, gynecology, and family planning. To be eligible, a clinician must be a physician, nurse practitioner, physician assistant, or nurse-midwife affiliated with study clinics and able to provide informed consent. Patient eligibility was having a clinician enrolled in the study, being aged 21 to 75 years, having no personal history of breast or ovarian cancer, having never received genetic counseling or testing for HBOC, meeting family history criteria for *BRCA1/2* genetic testing based upon a validated family history screener,^33^ and being able to provide informed consent in English or Spanish.

### Description of the Study Intervention

All participating patients received standard educational materials in English or Spanish, including a brochure from our institution's breast cancer prevention clinic and educational materials from the Susan G. Komen Foundation on genes and breast cancer. Additionally, all patients received a letter informing them of their eligibility for *BRCA1/2* genetic testing, outlining breast and ovarian cancer risk management options and encouraging them to discuss referral to genetic counseling with their clinician. Patients in the study intervention received the patient-facing RealRisks DA coupled with their clinicians receiving the BNAV tool.

Briefly, RealRisks is designed to improve a patient’s knowledge of breast cancer and personalized risk, as well as to support genetic testing decision-making. Upon logging into RealRisks, a patient is provided general education, and she is asked to enter her family history information into the tool. RealRisks then uses these data to calculate the patient’s breast cancer risk to provide personalized risk communication, tailored education, and decision support. Interactive games are used to communicate personalized 5-year and lifetime breast cancer risk and probability of carrying a *BRCA1/2* variant based on the BRCAPRO model.^52,53^ Education is provided as information-dense (ie, mainly text with definitions of medical terms) and information-light (ie, graphic novel narratives). Patient preferences are elicited using a slider tool. Women are also asked to indicate their intention to receive genetic testing. The patient’s risk, preference information, and intention are summarized for the patient in the patient action plan. Additional features to account for varying health and computer literacy include text to hover over to view definitions of key terms in the narrative, audio buttons, Spanish translations, and explanatory videos to navigate through the tool.

All clinicians had access to the BNAV tool through a link-out in the electronic health record (EHR) Ambulatory Medicine dashboard at CUIMC. BNAV consists of educational modules on breast cancer risk, genetic testing, and risk communication. For clinicians randomized to the intervention group, when their patients completed RealRisks, they were able to view personalized risk reports through a secure patient list within BNAV.^5^ The framework and data flow that enables RealRisks and BNAV to exchange data have been previously described.^54,55^

### Data Collection

Study questionnaires were administered to patients at baseline, 1 month, postclinic visit, and 6 months after randomization. The baseline questionnaire assessed sociodemographics (age, race and ethnicity, educational level, insurance, marital status) and validated measures for ehealth and health literacy,^56,57^ subjective numeracy,^58^ acculturation,^59^ control preferences in decision-making,^60^ and trust in clinicians.^61^ Decision-making measures to assess genetic testing knowledge,^62^ decision conflict,^63^ perceived breast cancer risk,^64^ breast cancer worry,^64,65^ and decision self-efficacy^66^ were measured at all time points. Genetic counseling and testing end points were assessed by EHR review and abstraction. Research staff conducted the abstraction using a structured database format following training by a senior study investigator (K.D.C.). Patient clinic visits with their enrolled clinician were assessed by patient self-report and confirmed by EHR review. RealRisks utilization was calculated by dividing the number of RealRisks modules completed by the number of modules required, as confirmed by examining server logs.

### Outcomes

The primary outcome was HBOC genetic counseling uptake within 6 months after enrollment, as assessed by EHR logs. Secondarily, we assessed receipt of genetic counseling at 24 months, genetic testing at 6 months and 24 months, and patient reports of genetic testing knowledge, breast cancer worry,^64,65^ decision self-efficacy,^66^ and decision conflict.^63^

### Statistical Analysis

A sample of 190 women, assuming a 20% dropout rate and an intracluster correlation of less than 0.2, was planned to provide 90% power at alpha = .05 2-sided significance level to detect a 25% difference in the primary outcome. We examined frequency distributions of baseline and demographic characteristics of participants and compared between the 2 groups using χ^2^ tests for categorical variables and *t* tests (or Wilcoxon rank-sum tests when nonparametric) for continuous variables. To compare 1- and 6-month survey variables between the groups and participant counseling and genetic testing uptake, we used linear regression models for continuous variables and simple or multiple logistic regression for categorical variables, with random effects to adjust for the clinician clusters. We calculated standard mean differences (SMDs) to estimate the effect of the intervention for continuous decision-making variables over time points. Data transformations were performed for skewed continuous outcomes, or nonparametric alternatives were used. These methods were also used to investigate the change in survey variables between baseline, 1 month, postclinic visit, and 6 months. For exploratory multivariate analyses investigating factors associated with EHR-confirmed genetic testing uptake, multilevel logistic regression models were fitted using generalized estimating equations with sandwich estimates of standard error and random effects adjustment for clinician clusters. Variables with a *P* < .10 in the bivariable tests were included in the models. Mean differences between randomization or tested and/or counseled groups were estimated with standard deviations, 95% CIs, and adjusted *P* values (to account for clustering by clinician). Overall, we found the intraclass correlation coefficient to be below 0.15. Odds ratios were generated for dichotomous outcomes, and 95% CIs were reported where appropriate. We analyzed data according to the intent-to-treat principle. Analyses were performed using SAS version 9.4 (SAS Institute) and R version 3.6.1 (R Project for Statistical Computing) from January to October 2021.

To ensure missing data did not bias our findings, we performed sensitivity analyses for our primary outcome variable using an imputed data set created with the R package MICE (Multivariate Imputation by Chained Equations) version 3.13. We assumed a monotone missing data pattern, used predictive mean matching for missing values, and conducted 10 iterations. We first performed this analysis only for participants with partially missing data at a follow-up time point. We then created another data set involving participants who were entirely missing a survey at a particular time point. Results for the imputed data sets were analyzed using the same methods as the primary data set, and then sensitivity analyses were conducted comparing against the primary data set.

## Results

Trial enrollment began in December 2018, and follow-up survey data collection ended in August 2020. A total of 190 patients enrolled in the trial under 67 clinicians (Figure). Of 187 evaluable patients enrolled (101 in the intervention group, 86 in the control group), 88 (46.6%) were Hispanic, 15 (8.1%) were non-Hispanic Black, 72 (38.9%) were non-Hispanic White; 35 (18.9%) had high school education or less, and 97 (51.9%) had private health insurance; the mean (SD) age was 40.7 (13.2) years (Table 1).^67^ Clinicians enrolled a range of 1 to 10 patients and a mean of 2.88 patients. Follow-up survey completion rates at 6 months were 85% in the intervention group and 88% in the control group. Demographics of participants who did not complete the 6-month survey were compared with participants completing the 6-month survey with no significant differences between groups. We also compared the demographics of participants who had a clinician visit with those not having a visit and found a significant difference in age, with older participants more likely to have a clinician visit.

**Figure.**
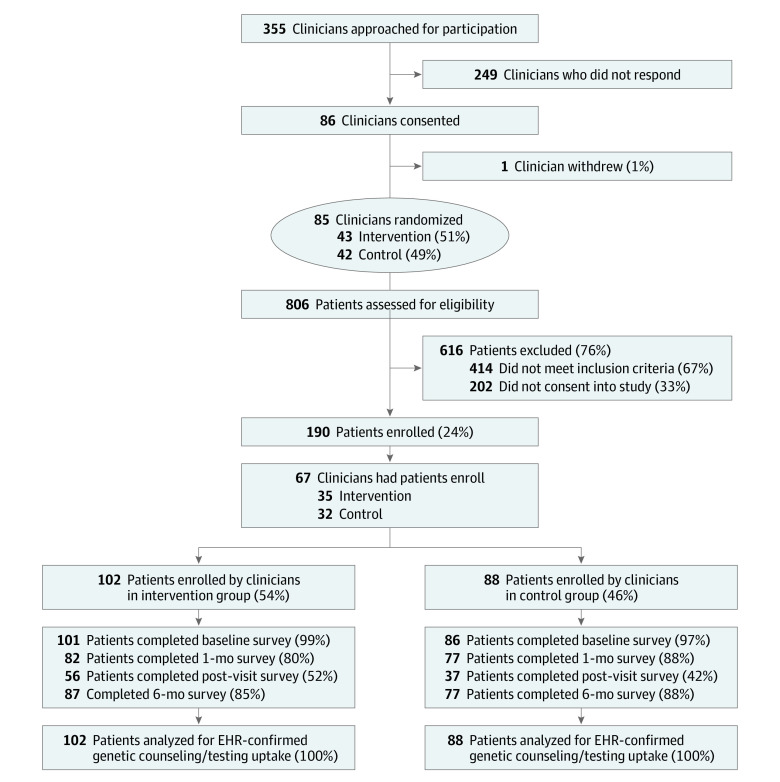
Study Flow Diagram EHR indicates electronic health record.

**Table 1.** Participant Characteristics at Baseline

Variable	No. (%)		
	Total (N = 187)	Intervention (n = 101)	Control (n = 86)
Age, mean (SD), y	40.7 (13.2)	41.3 (13.6)	40.0 (12.7)
Race and ethnicity			
Hispanic	88 (46.6)	48 (47.5)	40 (47.6)
Non-Hispanic			
Black	15 (8.1)	11 (10.9)	4 (4.8)
White	72 (38.9)	35 (34.7)	37 (44.0)
Other race and ethnicity^a^	10 (5.4)	7 (6.9)	3 (3.6)
Highest level of education			
High school or less	35 (18.9)	22 (22.0)	13 (15.3)
Some college or college degree	92 (49.7)	48 (48.0)	44 (51.8)
Postgraduate degree	58 (31.4)	30 (30.0)	28 (32.9)
Marital status			
Currently married	81 (43.5)	39 (38.6)	42 (49.4)
Single	78 (41.9)	45 (44.6)	33 (38.9)
Divorced or widowed	27 (14.5)	17 (16.8)	10 (11.8)
Health literacy [range: 0-4], mean (SD)^b^	1.6 (2.1)	1.7 (2.3)	1.5 (1.8)
Acculturation [range: 1-5], mean (SD)^c^	3.9 (1.5)	3.9 (1.5)	3.9 (1.4)
Subjective numeracy [range: 1-6], mean (SD)^d^	4.4 (1.1)	4.4 (1.1)	4.3 (1.2)
Health insurance			
Public (Medicaid, Medicare)	90 (48.1)	47 (46.5)	43 (50.0)
Private	97 (51.9)	54 (53.5)	43 (50.0)
6-point scale breast cancer risk [range: 1-40]^e^			
Mean (SD)	7.2 (3.3)	7.2 (3.4)	7.2 (3.2)
≤6	61 (32.6)	36 (35.6)	25 (29.1)
>6	126 (67.4)	65 (64.4)	61 (70.9)
Breast cancer worry [range: 2-14], mean (SD)^f^	6.5 (3.1)	6.6 (3.1)	6.3 (3.2)
Genetic testing knowledge^g^			
Adequate (≥ 7 correctly answered)	72 (38.5)	38 (37.6)	34 (39.5)
Inadequate (<7 correctly answered)	115 (61.5)	63 (62.4)	52 (60.5)
Genetic testing attitudes^h^			
Positive (≥ 25)	109 (58.6)	62 (61.4)	47 (55.3)
Negative (<25)	77 (41.4)	39 (38.6)	38 (44.7)
Decision self-efficacy [range 0-100], mean (SD)^i^	89.6 (13.2)	90.7 (10.7)	88.4 (15.5)
Control preference scale^j^			
Passive role	13 (7.0)	10 (9.9)	3 (3.5)
Collaborative role	84 (45.2)	45 (44.6)	39 (45.9)
Active role	89 (47.8)	46 (45.5)	43 (50.6)
Decision conflict [range: 0-100], mean (SD)^k^	42.5 (26.3)	41.1 (27.2)	44.2 (25.2)
Decision uncertainty [range: 0-100], mean (SD)^l^	40.8 (38.9)	40.6 (38.4)	41.0 (39.7)
Perceived 5-y risk [range: 0-100], mean (SD)^m^	47.7 (27.1)	47.5 (25.9)	48.0 (28.7)
Perceived lifetime risk [range: 0-100], mean (SD)^m^	31.5 (24.3)	33.8 (23.1)	28.7 (25.5)

^a^ Other race and ethnicity included Asian and unknown.

^b^ Health literacy^56^ three 5-point Likert scale measured using a validated scale to evaluate a person’s ability to read hospital materials alone, ability to understand medical conditions, and confidence in filling out forms alone. A higher score indicates lower health literacy.

^c^ Short Acculturation Scale for Hispanics^59^ evaluates a person’s preference between and usage of English and Spanish. A higher score indicates higher English acculturation.

^d^ Subjective numeracy scale^58^ estimates both risk comprehension and completion of utility elicitations without requiring survey participants to complete time-consuming and stress-inducing mathematics tests. A higher score indicates higher numeracy.

^e^ Six-point scale breast cancer risk is calculated based on a patient’s family history of breast cancer. A score higher than 6 indicates a high risk for breast cancer.

^f^ A higher worry score indicates higher worry about having breast cancer.

^g^ Genetic testing knowledge^62^ assessed by 11 items. Higher number of correct answers indicate more accurate knowledge.

^h^ Genetic testing attitude scale^67^ evaluates a patient’s attitudes about genetic testing. A higher score indicates more favorable attitudes.

^i^ Decision self-efficacy refers to a patient’s confidence in taking actions about breast cancer. A higher score indicates higher self-efficacy.

^j^ Control preference scale measures a patient’s preferred role in making treatment decisions with the help of clinicians.

^k^ Decision conflict measures a patient’s perception of factors contributing to choosing options. A higher score indicates higher decision conflict.

^l^ Decision uncertainty measures a patient’s perception of uncertainty in choosing options. A higher score indicates higher decision uncertainty.

^m^ Perceived risk measures a patient’s perceived chance of having breast cancer. A higher score indicates a higher perceived chance of having breast cancer.

### Primary Outcome

EHR-confirmed genetic counseling uptake within 6 months was not significantly different between the intervention and control groups. The intervention group had 20 patients (19.8%) with confirmed genetic counseling uptake vs 10 patients (11.6%) in the control group (difference, 8.2 percentage points; OR, 1.88; 95% CI, 0.82-4.30; *P* = .14) (Table 2).

**Table 2.** Primary and Secondary Genetic Counseling and Genetic Testing Comparisons

Characteristic	No. (%)		Adjusted odds ratio (95% CI)	*P* value
	Intervention (n = 101)	Control (n = 86)		
EHR-confirmed genetic counseling uptake				
Within 6 mo	20 (19.8)	10 (11.6)	1.88 (0.82-4.30)	.14
Within 24 mo	37 (36.6)	21 (24.4)	1.72 (0.84-3.54)	.14
EHR-confirmed genetic testing uptake				
Within 6 mo	13 (12.9)	7 (8.1)	1.73 (0.60-5.01)	.31
Within 24 mo	31 (30.7)	18 (20.9)	1.67 (0.85-3.29)	.14

### Secondary Outcomes

EHR–confirmed genetic testing within 6 months was also statistically nonsignificant (13 patients [12.9%] in the intervention group, 7 patients [8.1%] in the control group; difference, 4.8 percentage points; OR, 1.73; 95% CI, 0.60-5.01; *P* = .31). At 24 months, EHR-confirmed genetic counseling was received by 37 patients (36.6%) in the intervention group vs 21 patients (24.4%) in the control group (difference, 12.2 percentage points; OR, 1.72; 95% CI, 0.84-3.54; *P* = .14). Receipt of EHR-confirmed genetic testing at 24 months included 31 patients (30.7%) in the intervention group vs 18 patients (20.9%) in the control group (difference, 9.8 percentage points; OR, 1.67; 95% CI, 0.85-3.29; *P* = .14) (Table 2). Of the 187 women identified and enrolled, 58 participants (31.0%) underwent genetic counseling and 49 participants (26.2%) underwent genetic testing.

In terms of decision-making measures, decreases in breast cancer worry were greater in the intervention group from baseline to 6 months (SMD, −0.40) and from baseline to postclinic visit (SMD, −0.45) compared with the control group (Table 3). Genetic testing knowledge scores increased in both intervention and control groups; however, increases were significantly greater in the intervention group at baseline to 1 month (SMD, 0.35) and baseline to postclinic visit (SMD, 0.57). Perceived lifetime breast cancer risk decreased from baseline to post visit and to 6 months in the intervention group, but increased in the control group (SMD, −0.48). Women in both the intervention and control group reported lowered decision conflict and decision uncertainty at all time points, although differences between groups were not significant.

**Table 3.** Changes in Secondary Outcomes Over Time Points^a^

Variable	From baseline to 1 mo			From baseline to postclinic visit			From baseline to 6 mo		
	Mean (SD)		*P* value	Mean (SD)		*P* value	Mean (SD)		*P* value
	Intervention (n = 101)	Control (n = 86)		Intervention (n = 56)	Control (N = 37)		Intervention (n = 88)	Control (n = 75)	
Decision conflict [range, 0-100]	−11.4 (24.4)	−6.6 (24.7)	.28	−27.3 (26.8)	−22.7 (26.7)	.65	−19.3 (28.8)	−16.6 (28.7)	.81
Decision uncertainty [range: 0-100]	−6.6 (32.3)	−7.0 (40.8)	.82	NA	NA	NA	−18.5 (40.6)	−14.7 (39.2)	.73
Decision self-efficacy [range: 0-100]	1.6 (10.6)	0.4 (12.8)	.52	NA	NA	NA	0.0 (12.0)	−0.6 (13.8)	.89
Breast cancer worry [range: 2-14]	−0.7 (2.1)	−0.7 (2.1)	.07	−0.9 (2.7)	0.3 (2.3)	.04	−1.0 (2.7)	0.0 (2.3)	.01
Genetic testing knowledge [range: 0-22]	1.1 (2.3)	0.3 (2.3)	.03	1.0 (2.4)	−0.3 (2.3)	.03	0.6 (2.3)	0.1 (2.5)	.21
Attitudes Scale [range: 4-28]	−0.5 (4.0)	−1.1 (4.9)	.26	−0.3 (4.5)	−2.1 (5.2)	.06	−0.7 (5.0)	−0.8 (4.4)	.89
Perceived 5-y risk [range: 0-100]	−8.9 (21.8)	−2.5 (20.7)	.07	−9.3 (24/2)	−1.6 (21.3)	.26	−8.0 (21.0)	−2.7 (19.0)	.11
Perceived lifetime risk [range: 0-100]	−5.4 (21.0)	0.2 (19.3)	.09	−5.3 (24.2)	4.9 (17.9)	.04	−4.4 (20.1)	3.4 (19.9)	.02

Abbreviation: NA, not applicable.

^a^ The changes in continuous survey measures (5-year and lifetime risk, breast cancer worry, decision self-efficacy, decision conflict, decision uncertainty, knowledge, attitude) at the 3 time points were compared between the intervention and control groups, with adjustment for clinician clusters by using a mixed effects model. Positive values indicate an increase, negative values indicate a decrease.

### Exploratory Analysis: Factors Associated With Uptake of Genetic Testing

In bivariate analysis, race and ethnicity and age were the only baseline participant characteristics associated with uptake of genetic testing at 24 months (eTable in Supplement 2). However, when adjusting for other variables in the multivariate analysis, older age was the only baseline characteristic associated with genetic testing uptake (mean [SD] age, 45.1 [13.3] vs 39.2 [12.8]; OR, 1.04; 95% CI, 1.01-1.06; *P* = .01). In the multivariable model, genetic testing intention (OR, 3.01, 95% CI, 1.06-8.53; *P* = .03), RealRisks utilization (OR, 1.03; 95% CI, 1.00-1.07; *P* = .03), and genetic testing knowledge (OR, 1.28; 95% CI, 1.00-1.64; *P* = .047) were significantly associated with genetic testing uptake (Table 4).

**Table 4.** Multivariate Regression for 24-Month EHR-Confirmed Genetic Testing Uptake

Variable	Odds ratio (95% CI)	*P* value in type III test of fixed effects
Randomization group (intervention vs control)	0.09 (0.005-1.58)	.10
Decided to get testing at 6 mo (yes vs no)	3.01 (1.06-8.53)	.04
Clinic setting (private vs community)^a^	2.43 (0.62-9.45)	.20
Ever had clinic visit with clinician (yes vs no)	0.952 (0.339-2.67)	.92
RealRisks utilization^b^	1.03 (1.00-1.07)	.03
Attitude scores at 6 mo	1.12 (1.00-1.26)	.06
Knowledge at 6 mo	1.28 (1.00-1.64)	.05
Decision self-efficacy scores at 6 mo	1.01 (0.98-1.04)	.62
Age	1.04 (1.01-1.06)	.01
Race and ethnicity (minoritized racial and ethnic groups vs White)	1.18 (0.41-3.40)	.76
Health insurance type (private vs public)	1.26 (0.33-4.84)	.74
6-point scale risk scores (>6 vs ≤ 6)^c^	1.43 (0.65-3.14)	.37

^a^ Private clinic setting refers to the faculty practice at CUIMC, whereas the community clinic settings refer to an affiliated network of ambulatory care settings with emphasis on providing services regardless of inability to pay and usually takes care of Medicaid and Medicare patients.

^b^ RealRisks utilization is the percentage of completed modules in RealRisks.

^c^ Participants with a 6-point scale risk score greater than 6 had more red flags and therefore had a higher probability of being a *BRCA 1/2* carrier compared with participants with a score less than 6 assessed by the validated screener used in the study.

## Discussion

There was no statistically significant difference in genetic counseling uptake at 6 months, although uptake was 19.8% in the intervention group vs 11.6% in the control group. With respect to genetic testing uptake at 24 months, 30.7% of participants in the intervention group opted for genetic testing vs 20.9% in the control group, although differences between groups were also not statistically significant. With decision-making measures, participants in the intervention group reported statistically greater improvements in genetic testing knowledge at 1 month and postclinic visit, favorable genetic testing attitudes at postclinic visit, and decreased breast cancer worry at postclinic visit and 6 months. Other decision-making measures, such as decision conflict and decision uncertainty improved in both groups with no significant differences between groups.

Although we did not observe a significant difference between groups in genetic counseling uptake, of the women identified and enrolled, 58 of 187 participants (31.0%) underwent genetic counseling, and 49 of 187 participants (26.2%) underwent genetic testing. These results suggest important clinical significance, since without our intervention these women would likely not have been identified to enable comprehensive cancer risk management. Other studies seeking to identify individuals at risk for HBOC have relied on validated screening instruments. For example, Arun et al^68^ evaluated a screening tool to identify women who should be referred for genetic counseling.^68^ Among 1057 eligible patients, 142 (13.4%) attended a genetic counseling appointment. Similar studies to identify women at risk for HBOC in primary care clinics^69^ and mammography centers^70^ show comparable rates of uptake of cancer genetic counseling and testing (10%-20%). Genetic testing uptake was higher in our study compared with previous studies; however, additional research to further improve uptake and the health of women with hereditary cancer risk is warranted.

Clinician recommendation is often the first step toward uptake of genetic counseling, but a referral is less likely to occur among women in minoritized racial and ethnic groups and with patients with a lower level of education and limited health literacy.^28,33,34,71^ Even after a referral, genetic counseling remains underutilized and disparities with respect to uptake exist.^72^ In a study of 72 women with low income screened for family history, Pasick et al^73^ found only 4.5% uptake of a free genetic counseling session when patients were asked to schedule the appointment themselves. This finding suggests that removing cost barriers may be insufficient to maximize uptake of genetic counseling.

Cumulative evidence synthesized in a recent review suggests the importance of bidirectional influences in HBOC genetic counseling uptake, for example, among spousal and parent-child dyads.^74,75,76^ Decision-making about genetics is inherently different compared with other cancer screenings since genetic information can directly impact both the person undergoing testing and their biological family members. Yet, a recent systematic review found that resources to support decision-making about cancer genetic testing have an overemphasis on the cognitive aspects of making a decision (an individual-level factor) and lack tools that support patients through the decision-making process.^77^ Future research extending the use of DAs beyond the individual and clinician to better understand the family-based context in decisions to undergo genetic counseling and testing may be warranted.

An important difference between our study and previous work is that we assessed a racially and ethnically diverse patient population with a patient-facing DA, which is available in English and Spanish and has been rigorously tested among women of varying health literacy and numeracy.^48,49,50^ Additionally, our study simultaneously targeted both women and their clinicians.^51^ Our study reached a highly diverse population, with nearly half of the women recruited Hispanic and about 20% reporting a high school education or less. Reaching Hispanic women is vital because compared with non-Hispanic women, Hispanic women have lower awareness of genetic cancer risk assessment,^78^ lower testing uptake, and are more likely to have advanced-stage cancer at the time of diagnosis.^79,80,81,82,83,84^ In our study, we did not observe any racial and ethnic differences in genetic testing uptake, suggesting that DAs designed to be accessible to diverse populations and their clinicians play a role in ensuring equity of access among women eligible for HBOC genetic testing.^85^

Another strength of our study was the ability to track the actual use of the DA. We found among participants with EHR-confirmed genetic testing uptake, a higher level of RealRisks utilization was significantly associated with genetic testing uptake. This finding underscores the importance of DA utilization when they are implemented in clinical trials. Prior studies suggest that low fidelity to the intended use of DAs may underestimate their efficacy.^86,87,88,89^ Additional research is needed to assess factors that influence patient use and engagement and the true impact of DA implementation into clinical care.^89,90,91^ To increase RealRisks utilization, participants may have required additional engagement and support on how to access and navigate RealRisks. Therefore, we suggest this be addressed in future studies.

### Limitations

This study has limitations, including documentation of our genetic counseling and testing end points from the EHR of a single institution. Study participants may have received these services elsewhere leading to an underestimation of the study intervention. Second, we did not directly link patient access to RealRisks with the scheduled appointment with their clinicians, which may have further dampened the effect of the study intervention. Third, we were not able to track the use of BNAV by enrolled clinicians. Lack of use may have undermined the effectiveness of the intervention. Fourth, the trial completion rate was 85%. Fifth, the study was designed assuming what we considered to be a conservative estimate of 25% group difference, but the observed difference was only 8% in the primary outcome. This may reflect that both groups received robust interventions, and utilization of RealRisks was suboptimal.

## Conclusions

In this cluster randomized trial, we did not observe increased genetic counseling uptake with the intervention, but we did find statistically significant increases in genetic testing knowledge and decreased breast cancer worry and perceived risk. Although differences in uptake were not significant, more than one-third of women in the intervention group and nearly a quarter of women in the control group underwent genetic counseling. The main advantage for these high-risk women is the ability to opt for screening and preventive services to decrease their cancer risk. Future studies should focus on DA implementation into clinical care and promoting patient engagement and use of the DA. Although it is unlikely that there will be a one size fits all approach, DAs such as RealRisks may play a role in ensuring equity of access among diverse women eligible for HBOC genetic testing.

## Notes

Supplement 1.
Trial Protocol

Supplement 2. **eFigure.** Study Procedures **eTable.** Factors Associated With Genetic Testing Uptake, Bivariate

Supplement 3.
Data Sharing Statement
