# Supplementary material for: Patient and Clinician Decision Support to Increase Genetic Counseling for Hereditary Breast and Ovarian Cancer Syndrome in Primary Care: A Cluster Randomized Clinical Trial
Source: JAMA Netw Open. 2022 Jul 18;5(7):e2222092. doi: 10.1001/jamanetworkopen.2022.22092 (PMC9294997; doi:10.1001/jamanetworkopen.2022.22092)
Supplement: Supplement 1. — Trial Protocol [file jamanetwopen-e2222092-s001.pdf]

Trial Protocol

Background

Study Purpose and Rationale:

Hereditary breast and ovarian cancer syndrome (HBOC) is an inherited condition that is most commonly associated with mutations in the BRCA1 and BRCA2 genes. Mutation carriers have lifetime risks of breast and ovarian cancer of 40-60% and 20-40%, respectively. Risk management options include intensive breast cancer screening, risk-reducing surgeries, and chemoprevention, which have been shown to improve early detection and reduce cancer incidence and mortality. In particular, prophylactic bilateral salpingo-oophorectomy has been associated with a 60-77%reduction in all-cause mortality among BRCA mutation carriers. Based upon U.S. Preventive Services Task Force guidelines, an estimated 5% of women unaffected with breast cancer are eligible for genetic testing for HBOC, but only 14% of those eligible were referred and 4% had BRCA genetic testing. Many women may be unaware of their high-risk status due to our inability to adequately screen them in the primary care setting. Other reasons for low uptake include inadequate time for counseling and insufficient knowledge about risk-reducing strategies. Women from racial/ethnic minorities are less likely to seek genetic testing for HBOC, contributing to poorer clinical outcomes in these populations compared to non-Hispanic whites. Alternatively, decreasing inappropriate genetics referrals is also important for reducing anxiety and allowing genetic services to be used more efficiently. More research is needed to develop optimal strategies for engaging high-risk women in informed decision-making about genetic testing for HBOC.

The objective of this study is to expand genetic testing for HBOC to a broader population of high-risk women by prompting appropriate referrals from the primary care setting with the use of an electronic health record-embedded breast cancer risk navigation (BNAV) tool. To address patient-related barriers to genetic testing, we developed a web-based decision aid, RealRisks, which is designed to improve genetic testing knowledge, accuracy of breast cancer risk perceptions, and self-efficacy to engage in a collaborative dialogue about genetic testing.

Provide a description of how the confidentiality of study data will be ensured, addressing concerns or protections that specifically relate to the data storage elements identified above (e.g. hard copy, electronic, system, and/or endpoint): All subjects are assigned code numbers upon enrollment into the study. These codes are assigned to collected data and separated from a subject’s name or any other information that could identify them. The research files that link a subject’s name to the code number will be kept in locked file cabinets at the Department of Biomedical Informatics, and only the investigators and the study staff will have access to the files. All computer files will be protected by passwords available only to authorized study personnel. Electronic versions of consent forms and questionnaires will be administered by study staff and the data will be stored on our secure database server administered by the Department of Biomedical Informatics (system ID 5058), on the MC-domain shared drive administered by CUMC central IT (system ID 3959), and on a Filemaker database server

administered through the Department of Medicine (system ID 4029). Paper versions of CRFs are also transferred to the Velos Clinical Study Manager (system ID 3945) provided by the HICCC. Digital versions of the research files that link a subject's name to the code number are also kept in the certified systems referenced above. Only those individuals listed in the Personnel Section of this protocol will have access to these linking files. In addition, access to the BNAV tool for PCPs is through CUIT's Central Authentication Service (CAS), which uses the Columbia UNI. PCPs will only be able to see the health information of patients for whom they provide care.

Methodology:

We will conduct a cluster randomized controlled trial in the NYP/CUMC primary care and Ob/Gyn clinics. Providers from these clinics will be randomized to either an intervention or control arm. Patients will be assigned to the same arm as their provider. Participants in the control arm will receive educational brochures while those in the intervention arm will receive The primary endpoint is appropriate uptake of genetic counseling for HBOC syndrome by 6 months for the RealRisks plus BNAV arm vs. control arm.

Statistical Procedures:

The primary endpoint is appropriate uptake of genetic counseling for HBOC syndrome by 6 months for the RealRisks plus BNAV arm vs. control arm. We expect between 5-10% of women at the primary care clinics to be eligible for the study. Thus, we will enroll and screen 3,000 participants in order to accrue 200 eligible subjects. With a total sample size of 190 women eligible (95 per arm), assuming a two-sided Type 1 error of 5% and a 20% drop-out rate (effective sample size of 76 per arm), power calculations for a range of effect sizes are provided in Table 1. P0 and P1 are the rates of genetic counseling in the control and RealRisks/BNAV arms, respectively. The correlation coefficients adjust for clustering effect of the same physician treating multiple high-risk women. We will have >90% power to detect a difference of a 5% rate of appropriate genetic counseling in the control arm (based upon our pilot data) and 30% in the RealRisks + BNAV arm. Comparisons between the active and control groups will be conducted using Chi-square tests for categorical variables and Students t-tests for continuous variables. After generating descriptive statistics, we will conduct bivariate analyses using chi2 tests, t-tests, and Pearson correlation coefficients to determine associations between study variables and genetic counseling evaluation rate. Depending on the scale of each outcome variable (continuous or binary) and the scale of the independent variable (categorical or continuous), we will use ANOVAs, linear regression, logistic regression, variance components analysis, mixed models, and generalized estimating equations (GEE) to identify variables that are associated with each outcome. educational brochures coupled with RealRisks/BNAV intervention.

Privacy and Security:

Provide a description of how the confidentiality of study data will be ensured, addressing concerns or protections that specifically relate to the data storage elements identified above (e.g. hard copy, electronic, system, and/or endpoint):

All subjects are assigned code numbers upon enrollment into the study. These codes are assigned to collected data and separated from a subject's name or any other information that could identify them. The research files that link a subject's name to the code number will be kept in locked file cabinets at the Department of Biomedical Informatics, and only the investigators and the study staff will have access to the files. All computer files will be protected by passwords available only to authorized study personnel. Electronic versions of consent forms and questionnaires will be administered by study staff and the data will be stored on our secure database server administered by the Department of Biomedical Informatics (system ID 5058), on the MC-domain shared drive administered by CUMC central IT (system ID 3959), and on a Filemaker database server administered through the Department of Medicine (system ID 4029). Paper versions of CRFs are also transferred to the Velos Clinical Study Manager (system ID 3945) provided by the HICCC. Digital versions of the research files that link a subject's name to the code number are also kept in the certified systems referenced above. Only those individuals listed in the Personnel Section of this protocol will have access to these linking files.
In addition, access to the BNAV tool for PCPs is through CUIT's Central Authentication Service (CAS), which uses the Columbia UNI. PCPs will only be able to see the health information of patients for whom they provide care.

Description of the protections in place to safeguard participants' privacy while information is being collected.

For participants who are recruited in-person, they are approached and asked to participate in our study while they are at their primary care appointment at one of the clinic locations listed in the protocol. The discussion about the study and the informed consent process takes place inside of a private room that has been designated by the directors of each clinic.

Participants are asked survey questions directly by study staff while in the room or the participants are given the opportunity to fill out the surveys on their own either in the room or in the waiting area.

For participants who are recruited and complete study procedures over the phone, only staff who are listed in this protocol will have access to the study phone line and the study office. Research staff designated to collect consent will interact with participants over the phone in an office where 1-3 study personnel also sit.

Procedures:

Primary Care Providers (PCPs):

Potential PCP participants will be recruited from the CUMC/NYP Ambulatory Care Network (ACN) clinics and Columbia Doctors practices.

PCPs will be approached either in person, via mail, via email, or via phone. PCP recruitment flyers will also be posted at the clinics. The PCP's informed consent will be obtained by the research staff listed in the personnel section.

Eligibility criteria for PCPs include:

1) Primary care providers, such as attending physicians, residents, nurse practitioners, physician assistants, and midwives, who see patients at CUMC/NYP ACN clinics or a Columbia Doctors practice

2) Able to provide informed consent

After providing informed consent, PCP participants will complete a baseline questionnaire that collects information on their personal and professional characteristics, subjective risk communication confidence, confidence in managing patients with a family history of breast and ovarian cancer, knowledge, attitudes, orientation towards shared decision making, subjective norms, perceived behavioral control, and intention to refer high-risk patients to genetic testing. Primary care providers will be randomized to either the intervention or control arm.

PCPs in the intervention arm will then be given the Breast Cancer Risk Navigation Tool (BNAV), a web-based provider clinical decision support tool (https://kyras.dbmi.columbia.edu/bnav/), and will be asked to interact with the tool prior to the next clinical encounter with their participating patients. Through BNAV, the PCP will be able to access a patient's individualized risk profile, including family pedigree, and an educational module on genetic testing. The PCP will also be sent a copy of their patient's risk assessment information (BNAV PCP Action Plan) through email and a secure health message (SHM). The BNAV website will include a list of participating patients who are randomized to receive the intervention. The PCP will also be able to view a BNAV notice within the electronic health record (EHR) in iNYP, which includes the patient's personalized breast cancer risk profile and family pedigree.

PCPs in the control arm will not have access to a populated BNAV patient table, nor will they receive an action plan. These PCPs will, however, be able to view the BNAV notice within the electronic health record (EHR) in iNYP, which includes their patient's personalized breast cancer risk profile. PCPs will be asked another survey 6 months after baseline. This survey will assess confidence in managing patients with a family history of breast and ovarian cancer, knowledge, attitudes, orientation towards shared decision making, subjective norms, perceived behavioral control, and intention to refer the patient to genetic testing.

PCPs will also be asked to complete a survey after clinic visits with patients who are also participating in the research study. This survey will assess shared decision making.

Patients:

Participating PCPs' patients will also be recruited and will be pre-screened using a breast cancer risk survey including the Six-Point Scale family history screener to determine eligibility for genetic testing. If found eligible for the study, patient participants will participate in an informed consent process.

Patients will also have the option of self-referring themselves to the pre-screener. If a patient is found eligible, and her provider is not already enrolled in the study, we will recruit this provider as well. We will identify these providers either through a patient's self-report or from the EHR. After completing the prescreener survey, if a patient is eligible, a research team member will administer the BEATRICE Family History Intake Form over the phone or in person. The data collected by this form will flush out the data collected in the prescreener and provide more family history data that is needed for RealRisks to run its models. This information can also help us to verify that the patient is eligible.

Eligibility criteria for accrual of patient research participants into the cluster randomized controlled trial include:

1) Women, age 21-75 years without a personal history of breast or ovarian cancer
2) Meets criteria for genetic testing based upon the Six-Point Scale, defined in this study as 4 points or higher
3) No prior genetic counseling or testing for hereditary breast and ovarian cancer (HBOC)
4) Sees a primary care provider in the Columbia Univeristy/New York Presbyterian Hospital Ambulatory Care Network (ACN) or a Columbia Doctors practice
5) Understands as is willing to provide informed consent in English or in Spanish

Patient participants who enroll and complete the pre-screening questions will receive a risk notification indicating whether they are eligible for BRCA genetic testing. This risk message will be given to the participant either in-person, through mail, or by email.
Once patients have consented, we will check if they are registered to the clinic's Patient Portal. If not, we will work to register patient participants for the portal using the phone, email, in-person assistance, email invitations, and existing patient portal resources.
Upon meeting eligibility criteria and providing consent, all patient participants will be asked to complete the Baseline Survey. The baseline questionnaire assesses perceived risk and breast cancer worry, interest in genetic testing, health and eHealth literacy, decision self-efficacy, subjective numeracy, acculturation, preparation in shared decision making, decision conflict, knowledge, and attitudes by using validated and adapted measures. The baseline questionnaire is estimated to take 15-20 minutes and will be done either on paper, online, or over the phone. Patient participants will then receive an educational brochure for the CUMC Clinical Breast Cancer Prevention Program and a brochure that provides education on genetics and breast cancer. The risk notification and brochures will serve as the educational materials for the intervention and control arms. Those who do not meet high-risk criteria will be reassured that genetics referral is not indicated at this time, but should be reassessed if their personal or family history of cancer changes.

After completing the baseline questionnaire, patients in the intervention arm will also receive a link to the RealRisks decision tool. The research staff will determine the date and time of the patient’s next PCP appointment from the scheduling systems, the EHR, or the patient's self-report. Patients will be encouraged to use the tool within 2 weeks prior to their PCP visits and genetic counseling visits. Patients will be encouraged to bring a print-out of their action plan

(which will also be available to the PCP in the BNAV toolbox) based upon their interactions with the RealRisks decision aid, in order to facilitate discussions about genetic counseling and genetic testing. Control patients will be given access to and asked to complete RealRisks after they have completed all other study milestones and surveys.
All patients will be followed-up to complete a second survey within two months after they are given RealRisks and/or the control educational material. This survey assesses breast cancer worry, breast cancer risk perception, decision self-efficacy, knowledge, attitudes, preparation in decision making, decision conflict, and genetic testing decisions and asks for feedback on the intervention and/or control materials. The survey will be done either on paper, online, or over the phone. One week prior to an intervention-patient's schedule clinic visit with her enrolled-health care provider, she will be sent a prompt that will remind her of the information she entered into RealRisks and help her prepare for her appointment.

A survey will also be administered after a participant’s next scheduled appointment with her primary care provider. This survey will assess perceived risk and breast cancer worry, knowledge, attitudes, decision conflict, genetic testing decisions, shared decision making, trust in health care providers, and decisional regret. This survey will be distributed either on paper, online or over the phone. The clinic visit to which this survey refers will be the patients first visit after one month has passed since the patient was sent the high-risk letter/bochure/educational materials. If the patient is able to finish all preceding study milestones (baseline, RealRisks if in the intervention arm, and the 1-month survey) and then she has a clinic appointment before the one-month time point, this clinic visit can count. Additionally, if the patient missed or had to cancel the first visit after one month, we will use the next scheduled visit. If the patient and provider decide to speak more informally, for example over the phone, instead of hold a formal visit to discuss genetic counseling referral, we will count this as a visit.
Six months after patients are given RealRisks and/or the control materials, we will administer another follow-up survey. This survey will assess perceived risk and breast cancer worry, knowledge, attitudes, decision self-efficacy, decision conflict, genetic testing decisions, decisional regret, and genetic testing uptake. This survey will be distributed either on paper, online, or over the phone.

We will also assess for appropriate referrals to and uptake of genetic counseling and genetic testing after 6 months by using data from the EHR and scheduling systems.
To keep patient participants engaged throughout the research study, we may send calendars, thank-you cards, birthday cards, appointment reminders, and other similar retention materials. Providers will be sent post-it notes, thank-you cards, and other similar retention materials.
RETROSPECTIVE ANALYSIS OF ELECTRONIC HEALTH RECORDS:
We will also collect contact information, demographics, provider information, emergency contacts, breast imaging/pathology results, lab results, family history, reproductive history, clinical vitals (e.g., height, weight, BP), clinic encounters, insurance information, allergies, other breast cancer risk factors, medications, clinical notes, and clinical data related to other chronic diseases (such as cardiovascular disease and diabetes). We will assess referral and uptake of genetic counseling and genetic testing.

Chart Review of Electronic Health Records:

We will request a waiver of consent in order to collect similar EHR data on ACN patients who have not consented to enroll in the study protocol in order to identify potentially eligible women for recruitment. We will also use this data to assess the extent and quality of family history data in the EHR as well as other factors related to breast cancer genetic testing.

Recruitment:

Potential patient participants will be recruited from several different sources: KYRAS Parent Study (AAAO-1761), CUMC/NYP electronic health records (EHR), AVON mammography clinic, and from the community and clinics:

RECRUITMENT FROM THE KYRAS PARENT STUDY (IRB-AAAO1761), SISTER STUDY (IRB-AAAP4151), and investigator's other study (AAAJ9559)
Women that meet the eligibility criteria and gave permission for future research contact will be identified from KYRAS survey database (IRB-AAAO1761). They will be approached either in-person, via mail, via email, or via phone and study recruitment information will be given to them. If they are interested, they will be invited to participate in this study.
Potentially eligible patients identified through these study's EHR reviews/TRAC requests will also be noted, and their providers will be recruited. If these providers consent, we will send these patients initial contact messages and approach them if they do not opt out.

IN-PERSON RECRUITMENT IN CLINICS:
With the permission of the clinic's medical director or practice administrator, a member of the research team will wait in an area of the clinic designated by clinic staff with the Patient Flyer. Patients will be free to approach the team member for more information about the study. We will not approach patients ourselves unless they give permission to do so after their provider has introduced the study to them.

COMMUNITY RECRUITMENT
Recruitment flyers with eligibility criteria will be posted around the medical center campus, in the community, and online. Potential subjects will be directed to contact the study personnel through either phone or email or to the online pre-screener. We will also post recruitment flyers on the internet, using websites and platforms such as Instagram and facebook.

RECRUITMENT FROM AVON MAMMOGRAPHY CLINIC:
Our partner study (IRB: AAAP4151) will be recruiting patients from the AVON clinic. If a respondent to this questionnaire is found to be eligible for this research study, we will look to recruit her provider. Once her provider has consented, we will send the patient the initial contact message. If the patient doesn't opt out, she will be approached via phone, email, or in-person, and if interested, will be invited to participate in this study.

RECRUITMENT FROM ELECTRONIC HEALTH RECORDS AND SCHEDULING SYSTEMS:

Once a provider has consented, we will monitor the clinical scheduling systems for potentially eligible patients. These patients will be sent the "Initial Contact Message," and if they do not opt out, will be approached to participate in the study.

As only approximately 5% of the general population will be eligible for genetic testing, we will request TRAC requests in order to build a list of patients who are likely to be eligible for genetic counseling and, therefore, this study. We will use this list as a starting point from which to pre-screen patients. Additionally, if patients are identified as likely to be eligible for genetic counseling, and we do not have their providers enrolled, we will recruit their provider as well. We will collect contact information, demographics (for example, age, race, ethnicity, religion), provider information, emergency contacts, breast imaging/pathology results, lab results, family history, reproductive history, clinical vitals (e.g., height, weight, BP), clinic encounters (both past and scheduled), insurance information, allergies, other breast cancer risk factors, medications, clinical notes, and clinical data related to other chronic diseases (such as cardiovascular disease and diabetes).

We will also use OMPOP/ATLAS data to identify patients who are at an increased likelihood of being eligible for the study. Our goal is to predict breast cancer among the patients who have not yet been diagnosed of breast cancer and to prioritize the recruitment of these high-risk patients for the trial for cancer prevention because it is more likely that they will have a family history of breast/ovarian cancer. With the population EHR data from the institution, we can apply machine learning to build a prediction model to effectively identify the patients at high risk of breast cancer and, therefore, likely to be eligible for genetic testing and the study. We will be running the model for large-scale analysis, and we need a full population data in order to train our model on the population in its entirety for generalization. All patients in the database are candidates for the study, and we will use demographics (for example, age, race, ethnicity, religion), provider information, emergency contacts, breast imaging/pathology results, lab results, family history, reproductive history, clinical vitals (e.g., height, weight, BP), clinic encounters (both past and scheduled), insurance information, allergies, other breast cancer risk factors, medications, clinical notes, and clinical data related to other chronic diseases (such as cardiovascular disease and diabetes). These data will be used to phenotype and define a cohort of non-diagnosed, potentially high-risk patients as well as a cohort of breast cancer patients. The cohorts will then be used to develop and evaluate the prediction model. We require the data in an OMOP common data model for reproducibility of the model. For research purposes, we intend to build a prediction model based on the population data at our institution and also to reproduce and validate the model at external institutions who are collaborators in the OHDSI network using the OMOP common data model. OHDSI (Observational Health Data Sciences and Informatics) is an international collaborative research network that established a common data model (OMOP) to allow systemic analysis of observational databases. Only the algorithm will be shared to be reproduced and to be validated by external institutions with their own data in OMOP common data model, and our institution’s data will not be shared. We will also not be using data from other institutions. We also require the scheduling data to check whether high-risk patients have upcoming appointments with a study provider. We are only able to contact the patients with a consented provider, so we will first identify the high-risk patients using the

model, and will prioritize the recruitment to those who have an appointment scheduled with a consented provider. All patients who are not referred by their provider will continue to be sent the Initial Contact Message two weeks prior to being recruited. Patients will continue to be prescreened using our pre-screener for study recruitment.

RECRUITMENT PRIORITIZATION USING EMERGENCY CONTACT INFORMATION

Documentation of family history of breast or ovarian cancer in the EHR is commonly underreported. In order to obtain family history information so we can further prioritize our recruitment lists, we will utilize the method (RIFTEHR) developed by David Vawdrey and Nicholas Tatonetti (AAAO4154) to infer the presence of a family history using emergency contact linkages in the EHR. Our team will use RIFTEHR to flag patients whose emergency contacts have a history of breast or ovarian cancer and then prioritize reaching out to these patients. The members of the research team who contact patients will not have access to any information on the emergency contacts. They will only have a proband-level six-point scale estimation or a proband-level flag indicating a higher likelihood of a family history. We may also infer Ashkenazi Jewish ancestry using the method developed, which is an important factor included in the six-point scale estimation to indicate eligibility for BRCA genetic testing. This information, specifically a scoring from 0-6, will be the only information derived from this method that will be shown to recruiters to be used to prioritize our recruitment lists. Patients will still be sent the Initial Contact Message and will complete our family history pre-screener to determine eligibility before enrolling.

FOR PATIENTS WHO ARE IDENTIFIED THROUGH THE ELECTRONIC HEALTH RECORDS/SCHEDULING SYSTEMS OR AVON MAMMOGRAPHY CLINIC:

We will first contact the potential subject's treating physician or clinic director and request that the treating physician/clinic director sign the template of the "Initial Contact Message" that will be sent to the patient to introduce them to the study. Some providers will be asked to consent to this in their consent form.

If the provider/director signs off or consents, a signed letter or email will be sent to the patient with a brief overview of the study and request for permission to contact her for recruitment. The letter requests for patients to opt-out within two weeks if they do not wish to be contacted by the research team.

PATIENTS WHO ARE REFERRED TO THE STUDY BY THEIR PROVIDER:

If a provider introduces the study and the patient gives permission for us to contact them, we will also contact by phone or email.

SCREENING & CONSENT

Patients

When a potential human subject has been identified, the research staff will use a short script that briefly explains the study and eligibility criteria. If interested, we will continue to detail all

the steps of the study. We will then confirm the eligibility criteria. To assess for genetic counseling eligibility, we will use the Six-Point Scale, which has been adapted to reflect U.S. Preventative Services Task Force (USPSTF) guidelines and is an efficient tool developed in a population of ethnically diverse low-income women to determine eligibility for BRCA genetic testing.

If the participant meets study eligibility, they will be given the option to provide their informed consent over the phone, in-person, or online through Qualtrics. If consent is provided over the phone, the research staff will discuss the informed consent form fully with the participant and verbal consent will be obtained. A copy of the informed consent form will be sent to the participant. Participants who are consented in-person will be presented with the same consent forms, and their signatures will be obtained. If consented online, we will attempt to reach participants to review their consents; they will have the option of saving a copy of the consent directly from Qualtrics.

Potential patient participants will be encouraged to ask questions and their questions will be answered until the potential participants are satisfied. The voluntary nature of the study will be emphasized as well as the right to ask questions and withdraw at any time.

Primary Care Providers

Potential PCP participants will be recruited from the clinics. They will be approached either in person, via mail, via email, or via phone. PCP recruitment flyers will also be posted at the randomization sites. Some sites may distribute a description of the study in their internal newsletter.

Providers will be given the option to review the consent form online through Qualtrics, which will record the provider’s consent to participate. The provider will also be given the option to schedule a time to complete a verbal consent over the phone or an in-person appointment where written consent may be documented. For PCP participants consented by phone, the research staff will discuss the informed consent form fully with the provider and verbal consent will be obtained. Providers who are consented in-person will be presented with the same consent forms, and their signatures will be obtained. Potential PCP participants will be encouraged to ask questions and it will be explained that they can withdraw at any time.
In addition, the providers in the intervention arm will receive a provider alert ("BNAV Provider Email Alert") through a secure health message from the EHR and an email from the research staff which will include some of the patients' health information (PCP Action Plan) collected through this protocol (e.g. family history). The email and the alert will direct the provider to the Breast Cancer Navigation Tool (BNAV) which is intended for primary care providers. If the provider has not yet consented when accessing the BNAV tool, they will be presented with the option to consent online through Qualtrics. Provider participants are recruited because they are involved in the clinical encounter and care of the patients who are enrolled in the study.

Describe how participants' consent will be obtained and whether an information sheet will be used:

Verbal informed consent will be obtained and the research team member that obtains the informed consent will document it accordingly. When informed consent is obtained over the phone, we explain the study to the participant using the same language as that which is contained in the regular informed consent form that is being signed by those who attend in-person. This consent information will be used in an information sheet that will be provided to participant either through mail or at an in-person research appointment with the study. E-consent will be obtained from those consenting online and the participant will have the option of printing the consent form from the Qualtrics website.
For the usability sub-study, screening information will be collected before consent via the sub-study consent form.
Potential participants will first be verbally consented prior to the screening questionnaire using the
“Family History Intake Usability Sub-Study: Verbal Screening Consent Form"

Research Aims & Abstracts

Research Question(s)/Hypothesis(es):

Aim 1: To conduct a cluster randomized controlled trial of patient education with RealRisks plus BNAV compared to patient education alone among high-risk women identified in the primary care setting for appropriate uptake of genetic counseling.
Aim 2: To test whether patient education with RealRisks plus BNAV when integrated into clinical workflow decreases decisional conflict, improves shared decision-making, and increases informed choice about genetic counseling compared to using patient education alone.

Scientific Abstract:

The objective of this proposal is to expand genetic testing for hereditary breast and ovarian cancer syndrome (HBOC) to a broader population of high-risk women by prompting appropriate referrals from the primary care setting with the use of an electronic health record-embedded breast cancer risk navigation (BNAV) tool. To address patient-related barriers to genetic testing, we developed a web-based decision aid, RealRisks, which is designed to improve genetic testing knowledge, accuracy of breast cancer risk perceptions, and self-efficacy to engage in a collaborative dialogue about genetic testing. We hypothesize that combining a patient-centered decision aid with a provider-centered decision support tool integrated into clinic workflow will increase appropriate uptake of genetic counseling. We also hypothesize that genetic counseling decisions will be more informed, and result in less decision conflict and improved shared decision making.

High-risk women (N=200) will be screened in mammography and gynecology clinics by patient recruiters administering the Six-Point Scale, an efficient family history screener, to determine

eligibility for genetic counseling. We will randomize primary care providers to patient education plus the web-based RealRisks in combination with BNAV (group 1) vs. patient education alone (group 2). Assignment of high-risk women to the interventions will be based upon primary care provider. The primary endpoint is appropriate uptake of genetic counseling by 6 months.

This study seeks to overcome important patient and provider-related barriers to appropriate uptake of genetic counseling among racially/ethnically diverse high-risk women identified in the primary care setting. We seek to evaluate a more efficient informatics-driven model for screening high-risk women and administering genetic testing services. With appropriate use of genetic counseling for HBOC, our goal is to improve cancer risk assessment and enhance uptake of risk-appropriate screening and prevention strategies.

Lay Abstract:

The objective of this proposal is to expand genetic testing for women who are at higher risk of breast and ovarian cancers because of their family history and genetics. We propose to promote referrals to genetic counseling by primary care providers (PCP) in order to increase the amount of women at higher risk who end up getting genetic testing. We intend to increase referrals in primary care clinics by using a patient-centered website called RealRisks and PCP-centered website called BNAV.

RealRisks addresses patient-related barriers to genetic testing and assists patients in making and informed decision regarding their breast health. It is designed to improve genetic testing knowledge, accuracy of breast cancer risk perceptions, and self-efficacy to engage in conversation with their primary care providers regarding genetic testing.
We believe that combining a patient-centered decision aid with a provider-centered decision support tool integrated into clinic workflow will increase appropriate uptake of genetic counseling. We also hypothesize that genetic counseling decisions will be more informed, and result in less decision conflict and improved shared decision making.

High-risk women (N=200) will be screened by patient recruiters administering the Six-Point Scale, a family history questionnaire, to determine eligibility for genetic counseling. We will assign the RealRisks and BNAV websites combined with educational materials as the intervention arm (group 1), and the control arm will simply be educational materials (group 2). We will randomly assign providers to one of the two groups, and all of a provider’s patients will be assigned to his or her same group. The main outcome is appropriate uptake of genetic counseling by 6 months.

We will assess the effectiveness of the RealRisks/BNAV intervention with assessments of the process by which the decision was made and rating the quality of that decision. The secondary outcome that will be analyzed is the degree of decisional conflict, a key predictor of decision making, which is defined as “personal uncertainly about which option to choose”. To evaluate whether participants made an informed choice, patients will be classified as having made an informed or uninformed choice.

This study seeks to overcome important patient and provider-related barriers to appropriate uptake of genetic counseling among racially/ethnically diverse high-risk women identified in the primary care setting. We seek to evaluate a more efficient informatics-driven model for screening high-risk women and administering genetic testing services. With appropriate use of genetic counseling for HBOC, our goal is to improve cancer risk assessment and enhance uptake of risk-appropriate screening and prevention strategies.

Potential Risks:

Provide information regarding all risks to participants that are directly related to participation in this protocol, including any potential for a breach of confidentiality. Risks associated with any of the items described in the Procedures section of this submission should be outlined here if they are not captured in a stand-alone protocol. Risks of procedures that individuals would be exposed to regardless of whether they choose to participate in this research need not be detailed in this section, unless evaluation of those risks is the focus of this research. When applicable, the likelihood of certain risks should be explained and data on risks that have been encountered in past studies should be provided.

Potential Benefits:

The direct benefits associated with participating in this study include increased knowledge about the subject's personal breast and ovarian cancer risk, as well as the opportunity to learn about strategies to minimize their future risk. The information the subjects provide will also help researchers better understand decision-making regarding genetic testing among high-risk women. Future generations may benefit from the results and knowledge gained from this study. As a result, patients may gain a sense of well-being from this knowledge.

Subject Population Justification:

Although the prevalence of BRCA mutations is similar across U.S. ethnic groups (except for the Ashkenazi Jewish population), genetic testing is less likely to occur among non-white women and those with lower educational and income levels. Among Hispanics, who represent the largest racial/ethnic minority (17.4%) in the U.S., breast cancer is the most common malignancy and the leading cause of cancer-related death among women. Women from racial/ethnic minorities are less likely to seek breast cancer preventive care, contributing to higher rates of late stage diagnosis and poorer clinical outcomes in these populations compared to non-Hispanic whites. Since this study involves patient-provider dyads, we will also include these women's primary care providers at the Columbia University Medical Center/NYP.

Reimbursement/compensation:

The patient participant will receive $25 for the baseline survey, $25 for the two-week survey, $50 for the six-month survey, and $50 for the survey after the next clinical appointment with

their primary care provider. Patient participants will also receive $20 for completing the RealRisks decision aid and $5 for scheduling an appointment with their enrolled health care provider.

The provider participant will receive $50 for the baseline survey, $50 for the second survey, and $50 for each post-encounter survey.
