# Supplementary material for: Patient and Clinician Decision Support to Increase Genetic Counseling for Hereditary Breast and Ovarian Cancer Syndrome in Primary Care: A Cluster Randomized Clinical Trial
Source: JAMA Netw Open. 2022 Jul 18;5(7):e2222092. doi: 10.1001/jamanetworkopen.2022.22092 (PMC9294997; doi:10.1001/jamanetworkopen.2022.22092)
Supplement: Supplement 2. — eFigure. Study Procedures eTable. Factors Associated With Genetic Testing Uptake, Bivariate [file jamanetwopen-e2222092-s002.pdf]

Supplemental Online Content

Kukafka R, Pan S, Silverman T, et al. Patient and clinician decision support to increase genetic counseling for hereditary breast and ovarian cancer syndrome in primary care: a cluster randomized clinical trial. *JAMA Netw Open.* 2022;5(7):e2222092. doi:10.1001/jamanetworkopen.2022.22092

eFigure. Study Procedures

eTable. Factors Associated With Genetic Testing Uptake, Bivariate

This supplemental material has been provided by the authors to give readers additional information about their work.

© 2022 Kukafka R et al. *JAMA Network Open.*

eFigure. Study Procedures

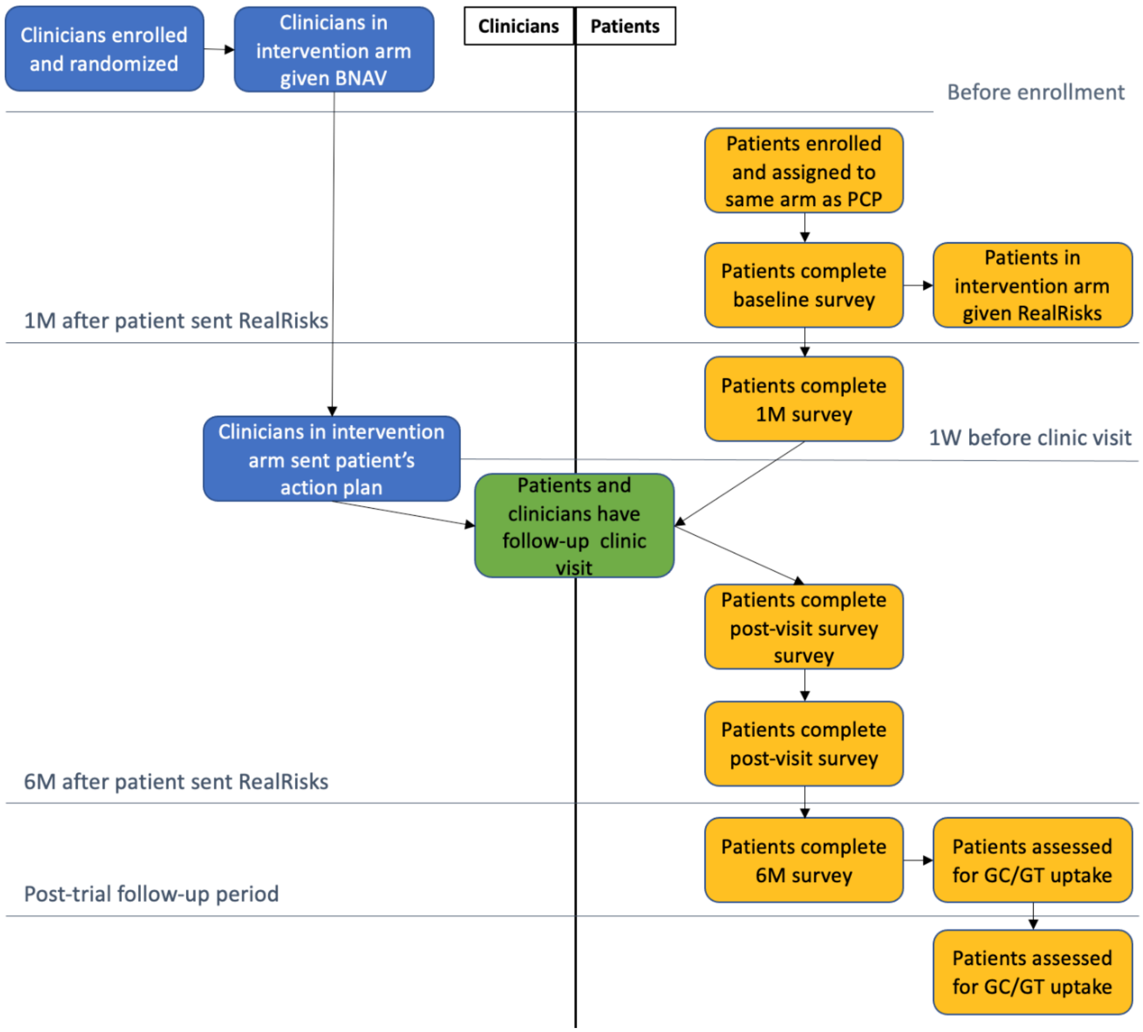

© 2022 Kukafka R et al. *JAMA Network Open.*

eTable. Factors Associated With Genetic Testing Uptake, Bivariate

Variable, N (%) or Mean (SD)	Patients with documented genetic testing at post-trial follow up (n = 49)	Patients without documented genetic testing at post-trial follow up (n = 138)	Difference of mean change (95% CI)	Adjusted Odds Ratio (95% CI)	Adjusted P value
Randomization arm	n = 49	n = 138			
Intervention, N (%)	31 (63.3)	70 (50.7)	NA	1.67 (0.85-3.29)	0.13[a]
[Control], N (%)	18 (36.7)	68 (49.3)	NA		
Clinic setting [a]	n = 49	n = 138			
Private practice, N (%)	33 (67.3)	69 (50.0)	NA	2.08 (1.04-4.18)	0.04
Community clinic, N (%)	16 (32.7)	69 (50.0)	NA		
Clinician type	n = 49	n = 138			
OB/GYN, N (%)	27 (55.1)	74 (53.6)	NA	1.11 (0.57-2.16)	0.77
[Other], N (%)	22 (44.9)	64 (46.4)	NA		
Ever had clinic visit with clinician	n = 49	n = 138			
Yes, N (%)	33 (67.3)	60 (43.5)	NA	2.59 (1.29-5.19)	0.01
[No], N (%)	16 (32.7)	78 (56.5)	NA		
*RealRisk*s utilization (%)	n = 31	n = 68			0.05
Mean (SD)	97.7 (5.4)	77.8 (37.3)	-19.9 (-29.1- -10.6)	1.05 (1.00-1.09)	
Genetic testing intention at 6 months	n = 49	n = 138			
Decided to get testing, N (%)	38 (77.6)	61 (44.2)	NA	4.78 (2.10-10.9)	< .001
[Other], N (%)	11 (22.4)	77 (55.8)	NA		
Decision conflict scores at 6 months	n = 48	n = 115			0.01
Mean (SD)	15.1 (17.5)	28.6 (26.2)	13.5 (6.6-20.5)	0.974 (0.957-0.991)	
Decision self-efficacy scores at 6 months	n = 48	n = 115			0.04
Mean (SD)	93.0 (10.1)	87.9 (14.5)	-5.1 (-9.1- -1.2)	1.04 (1.00-1.07)	
Attitudes scores at 6 months	n = 48	n = 113			< .001
Mean (SD)	25.1 (3.6)	22.7 (5.2)	-2.4 (-3.8- -1.0)	1.14 (1.04-1.26)	
Knowledge at 6 months	n = 48	n = 112			0.01
Mean (SD)	7.1 (2.2)	5.7 (2.6)	-1.6 (-2.3- -0.6)	1.29 (1.10-1.52)	
Worry scores at 6 months	n = 48	n = 113			
Mean (SD)	5.6 (2.7)	6.0 (2.8)	0.4 (-0.5,-1.4)	0.955 (0.836-1.09)	0.50
Age	n = 49	n = 138			0.01
Mean (SD)	45.1 (13.3)	39.2 (12.8)	-5.9 (-10.1- -1.6)	1.03 (1.01-1.06)	
Race/Ethnicity [b]	n = 48	n = 137			
White/non-Hispanic, N (%)	24 (50.0)	48 (35.0)	NA	0.501 (0.252-0.994)	0.05[a]
Other, N (%)	24 (50.0)	89 (65.0)	NA		
Health literacy scores	n = 49	n = 138			
Mean (SD)	1.9 (2.8)	1.5 (1.8)	-0.4 (-1.1-0.23)	1.09 (0.94-1.27)	0.26
Subjective Numeracy	n = 49	n = 138			
Mean (SD)	4.5 (1.1)	4.3 (1.1)	-0.2 (-0.6-0.2)	1.16 (0.85-1.58)	0.35

© 2022 Kukafka R et al. *JAMA Network Open.*

eHealth Literacy 1	n = 49	n = 138			
Mean (SD)	3.8 (1.0)	3.8 (0.9)	-0.0 (-0.2, 0.4)	0.958 (0.683-1.35)	0.80
eHealth Literacy 2	n = 49	n = 138			
Mean (SD)	4.0 (0.9)	4.2 (0.8)	0.2 (-0.1, 0.5)	0.803 (0.547-1.18)	0.26
Health insurance type	n = 49	n = 138			
Private, N (%)	30 (61.2)	67 (48.6)	NA	1.66 (0.84-3.25)	0.14
[Public], N (%)	19 (38.8)	71 (51.4)	NA		
Control preference scale	n = 49	n = 137			
[Passive role], N (%)	3 (6.1)	10 (7.3)	NA		0.45
Collaborative role, N (%)	19 (38.8)	65 (47.4)	NA	1.10 (0.27-4.52)	0.90
Active role, N (%)	27 (55.1)	62 (45.3)	NA	1.66 (0.41, 6.71)	0.47
Six-point scale risk scores [c]	n = 49	n = 138			
Mean (SD)	7.8 (3.2)	7.0 (3.3)	-0.8 (-1.9, 0.3)	1.07 (0.97-1.18)	0.16
[< 6], N (%)	12 (24.5)	49 (35.5)	NA	1.78 (0.84, 3.78)	0.13
>= 6, N (%)	37 (75.5)	89 (64.5)	NA		

[a] "private" clinic setting refers to the faculty practice at CUIMC, whereas the "community" clinic settings refer to an affiliated network of ambulatory care settings with emphasis on providing services regardless of inability to pay.

[b] categories of racial/ethnic diversity (table 1) vs. non-Hispanic white.

[c] participants with a six-point scale risk score <= 6) had more "red flags" and therefore had a higher probability of being a BRCA1/2 carrier compared to participants with a score >6 assessed by the validated screener used in the study.

[d] brackets indicate the reference group for odds ratio comparison in this categorical variable.

[e] odds ratio is for each unit increase in this continuous variable/scale.

© 2022 Kukafka R et al. *JAMA Network Open.*
