# Supplementary material for: Patient and Clinician Decision Support to Increase Genetic Counseling for Hereditary Breast and Ovarian Cancer Syndrome in Primary Care: A Cluster Randomized Clinical Trial
Source: JAMA Netw Open. 2022 Jul 18;5(7):e2222092. doi: 10.1001/jamanetworkopen.2022.22092 (PMC9294997; doi:10.1001/jamanetworkopen.2022.22092)
Supplement: Supplement 3. — Data Sharing Statement [file jamanetwopen-e2222092-s003.pdf]

**Data Sharing Statement**

Kukafka R, Pan S, Silverman T, et al. Patient and clinician decision support to increase genetic counseling for hereditary breast and ovarian cancer syndrome in primary care. *JAMA Netw Open*. 2022;5(7):e2222092. doi:10.1001/jamanetworkopen.2022.22092

**Data**
**Data available:** Yes
**Data types:** Deidentified participant data
**How to access data:** Data requests to Rita Kukafka rk326@cumc.columbia.edu
**When available:** With publication

**Supporting Documents**
**Document types:** None

**Additional Information**
**Who can access the data:** Rita Kukafka rk326@cumc.columbia.edu
**Types of analyses:** For any purpose
**Mechanisms of data availability:** with a signed data access agreement.
